# Synthesis of Co–Fe 1D Nanocone Array Electrodes Using Aluminum Oxide Template

**DOI:** 10.3390/ma14071717

**Published:** 2021-03-31

**Authors:** Katarzyna Skibińska, Karolina Kołczyk-Siedlecka, Dawid Kutyła, Marta Gajewska, Piotr Żabiński

**Affiliations:** 1 Department of Physiochemistry and Metallurgy of Non-Ferrous Metals, Faculty of Non-Ferrous Metals, AGH University of Science and Technology, A. Mickiewicza 30, 30-059 Krakow, Poland; kkolczyk@agh.edu.pl (K.K.-S.); kutyla@agh.edu.pl (D.K.); zabinski@agh.edu.pl (P.Ż.); 2 Academic Centre for Materials and Nanotechnology, AGH University of Science and Technology, A. Mickiewicza 30, 30-059 Krakow, Poland; marta.gajewska@agh.edu.pl

**Keywords:** 1D nanostructures, AAO template, Co–Fe alloy, hydrogen evolution

## Abstract

Porous anodic alumina oxide (AAO) obtained via two-step anodization is a material commonly used as a template for fabricating 1D nanostructures. In this work, copper and cobalt-iron 1D nanocones were obtained by an electrodeposition method using AAO templates. The templates were produced using two-step anodization in H_2_C_2_O_4_. The Co–Fe nanostructures are characterized by homogeneous pore distribution. The electrocatalytic activity of the produced nanomaterials was determined in 1 M NaOH using the linear sweep voltammetry (LSV) and chronopotentiometry (CP) methods. These materials can be used as catalysts in the water-splitting reaction. The sample’s active surface area was calculated and compared with bulk materials.

## 1. Introduction

Anodization is a widely used surface treatment method to increase the mechanical and tribological properties [1,2,3,4,5,6,7,8] and corrosion protection [9,10] of aluminum [11] and its alloys [12]. It can also be used as a template for the fabrication of micro and nanostructures [13,14]. Porous anodic alumina oxide (AAO) is characterized by a honeycomb structure, a close-packed hexagonal array of parallel cylindrical nanopores perpendicular to the surface of the aluminum [15].

The porous anodic alumina oxide template is widely used in different sectors, such as nanofabrication [16,17,18], bioengineering [19,20], anti-reflection sheets [21,22], surface-enhanced Raman spectroscopy [23,24] and superhydrophobic surfaces [25]. Application in the optical domain [26] is connected with different values of the effective refractive index of AAO due to its porosity [27].

A surface is called superhydrophobic when its water contact angle is higher than 150° [28]. There are two significant factors connected with this phenomenon: surface energy and surface morphology [29]. Interest in the application of superhydrophobic structures increased due to their excellent properties, such as anti-corrosion [28,30] and self-cleaning [31]. The conical shape of the structures ensures the superhydrophobic properties of the material [29,32,33,34]. Nanocones obtained using AAO have potential applications connected with this property.

Two-step anodization is commonly used, due to the simplicity and low cost of the process, for the synthesis of well-ordered and high-quality 1D nanostructures, such as nanowires [16,28,29,30], nanodots [35], nanocones [17] and nanotubes [36]. An advantage of this 1D material is its improved catalytic performance compared with bulk materials due to its large active surface area and small geometrical size [17]. This phenomenon has been observed for 1D nanocone-structured electrodes. In comparison with nanowires, nanocones stand up straight and do not tend to bend or fall.

In recent years, attention to the application of hydrogen as a fuel in the renewable energy field increased [37,38]. The water-splitting reaction allows for obtaining hydrogen characterized by a high purity. This reaction enables the reduction of costs and energy due to cheap electrodes with low overpotential for hydrogen evolution [39,40,41]. This overpotential can be reduced by producing nanostructures that increase the active surface area [17].

Obtaining templates for the fabrication of nanocones consists of alternating anodization and pore-widening processes in the second step of anodization. The two-electrode system is used, where the cathode is Pt and the anode is Al.

Brzozka et al. [17] sputtered a thin, conductive copper layer on a prepared AAO nanocone template using a vacuum sputter coater (Emitech K575X). They then performed direct current (DC) electrodeposition of this metal in a three-electrode cell. The deposition was carried out at room temperature at 5 mA/cm^2^ for 60 min in an aqueous solution, which contained 0.5 M CuSO_4_ and 0.5 M H_2_SO_4_. To obtain a free-standing Cu nanocone array, the Al_2_O_3_ template was immersed into a dilute phosphoric acid solution.

Alternatively, Tagaura et al. [42] deposited electroless Ni on an AAO 1D template. They applied pulsed palladium electrodeposition in a PdCl_2_–HCl solution to produce small Pd particles, which took part in the Ni deposition on the template. The electroless deposition was performed for several minutes. To obtain a thicker layer, Ni electrodeposition was conducted on the deposited Ni layer from a Watts bath using a three-electrode cell, a Pt sheet as a working electrode and an Ag or AgCl electrode as a reference electrode.

Co–Fe alloys are characterized by high Curie temperatures (>1093 K), the highest magnetic saturation [43] and their ability to be easily obtained by electrodeposition from aqueous solutions [44]. An applied magnetic field during electrodeposition influences the morphology of the obtained coatings [45]. In the Co–Fe phase diagram, the ordered B2 phase is formed for the alloy composition between 25–72% at. Co [46]. Cobalt, like nickel and iron, is usually used as a material for electrodes in the alkaline environment due to its low price and appropriate physicochemical properties. Co–Fe alloys can be characterized by high catalytic activity and corrosion resistance. The literature research showed that Fe–Co alloy nanoparticles exhibited excellent electrocatalytic hydrogen activity [47]. This suggests that Co–Fe alloy nanocones can potentially be applied as catalysts in water-splitting reactions as a substitution for expensive noble metals. However, there are no works connected with the synthesis of Co–Fe conical structures using AAO.

In this work, copper and cobalt–iron alloys were electrodeposited using a nanocone AAO template. The Co–Fe alloys with uniform distribution of these elements were successfully obtained by electrodeposition using AAO. We attempted to establish the electrocatalytic properties of the 1D conical structures of cobalt–iron alloys. The second step was a comparison of 1D structures, with electrodeposited bulk with the same composition to test the scale of the increase of electrocatalytic activity of the 1D nanostructures.

## 2. Materials and Methods

### 2.1. Fabrication of AAO Templates

Firstly, an aluminum (AA 1050) sample was electropolished using a Struers LectroPol-5 (Copenhagen, Denmark). Then, the first step of the anodization process was performed using 0.3 M H_2_C_2_O_4_ at 2 °C and 45 V for 60 min. This step is called long-period anodization. The obtained oxide layer was removed by immersion into a mixed solution of 1.8 wt.% chromic and 6 wt.% phosphoric acid. The second step of anodization consisted of four short anodization cycles in 0.3 M H_2_C_2_O_4_ at 9 °C and at 45 V (for 25 s in the first cycle and 20 s for the next cycles) and a pore widening process in 5 wt.% H_3_PO_4_ at 30 °C for 12 min. The terms long-period and short-period anodization are used to highlight the difference in duration of these processes. The second step of anodization was also performed at the higher temperature. All reagents were characterized by analytical purity (POCH S.A., Gliwice, Poland). Deionized water was used to prepare the solutions.

### 2.2. Electrodeposition of Metal and Alloys

To maintain the conductivity of the sample, a porous anodic alumina oxide template with a sputtered thin copper layer was used. Electrodeposition of the Cu and Co–Fe alloys was then performed using templates obtained in oxalic acid. In all cases, porous anodic alumina oxide was the cathode, and platinum foil was the anode.

Electrodeposition of the Cu was performed for 2 h in a two-electrode cell at room temperature with AAO as the cathode and Pt as the anode. The applied current density was equal to 5 mA/cm^2^. The electrodeposition was performed from an aqueous electrolyte containing 0.5 M CuSO_4_ and 0.5 M H_2_SO_4_.

Co–Fe bulk coating and Co–Fe nanoconical structures were electrodeposited in potentiostatic measurements (−1.285 V vs. the saturated calomel electrode (SCE)). Each sample was deposited from electrolytes with the composition 6.5 mM CoSO_4_, 1.6 mM FeSO_4_ and 98.4 mM Na_2_SO_4_ for the first coating and 6.5 mM CoSO_4_, 3.3 mM FeSO_4_ and 96.8 mM Na_2_SO_4_ for the second coating and nanocones [36]. Electrodeposition was performed in a three-electrode cell at room temperature with a Cu plate as the cathode, Pt as the anode and saturated calomel electrode (SCE) as the reference. All the experiments took 2 h. The cathodic and the anodic parts of the cell were separated to avoid any undesired changes in the oxidation state of Fe^2+^ and Fe^3+^ ions.

To obtain free-standing structures, the templates were removed by immersion into a 5 wt.% H_2_SO_4_ solution in the case of the Cu nanocones and a diluted NaOH solution for the Co–Fe nanocones. This allowed avoiding the dissolving of the alloy components.

### 2.3. Microstructal Charaterization

**Figure 1:**
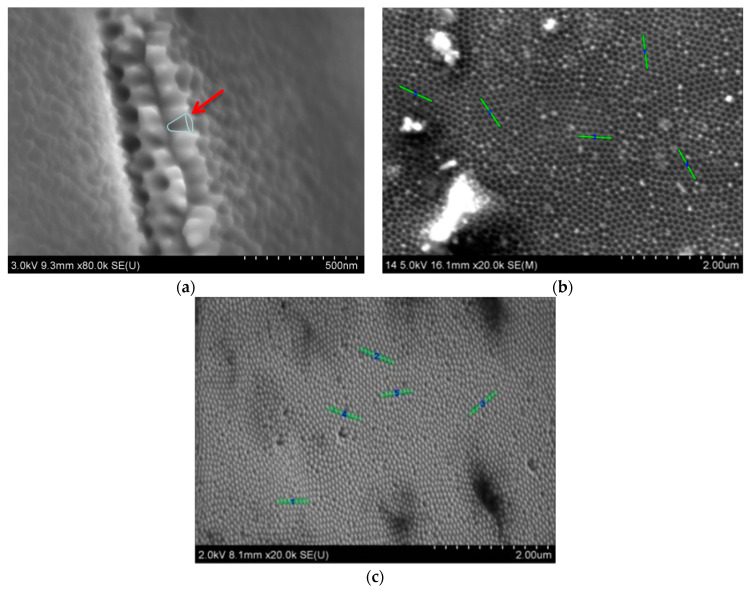
The comparison of nanopore and nanocone average distances, determined using SEM photos of (**a**) a cross-sectional view of the Al_2_O_3_/Al template obtained after four alternating anodizing–etching cycles in 0.3 M H_2_C_2_O_4_ and 45 V at 2 °C; (**b**) a top view of the same template and (**c**) a top view of free-standing copper nanocones.

Elemental analysis of the electrodeposited Co–Fe coatings was performed using the WD X-ray fluorescence (XRF) method (RigakuPrimini). The microstructures and the distributions of the nanocones were observed using the SEM technique (Jeol JCM-6000 Plus Versatile Benchtop SEM, Tokyo, Japan) and energy-dispersive X-ray spectroscopy (EDS) (Tokyo, Japan) analysis. The SEM images showed in Figure 1 were taken through a Hitachi SU-70 scanning electron microscope (Tokyo, Japan). The surface of the Cu conical nanostructures was analyzed using the atomic force microscope (AFM) Ntegra Aura microscope (NT MDT, Moscow, Russia) with an NSG03 tip.

Transmission electron microscopy (TEM) investigations were carried out using an FEI Tecnai TF20 X-TWIN (FEG) microscope (Tokyo, Japan) equipped with an energy-dispersive X-ray spectrometer (EDAX), working at an accelerating voltage of 200 kV.

Thin foils for the TEM investigations were prepared via the focused ion beam (FIB) technique with an FEI Quanta 3D 200i FIB/SEM dual-beam microscope (Tokyo, Japan) equipped with an OmniProbe micromanipulator. An ion beam accelerating voltage of 30 kV and ion currents in the range of 32–0.05 nA were applied.

### 2.4. Electrochemical Characterization

The electrocatalytic activity of each sample was determined in 1 M NaOH in a three-electrode cell. A bulk or nanocone electrode was the working electrode, a Pt foil was the anode and a saturated calomel electrode (SCE) was the reference electrode. The linear sweep voltammetry (LSV) measurements were performed in a range from the value of the open circuit potential (OCP) to −2 V vs. the SCE with a scan speed equal to 5 mV/s in the non-stirred electrolyte. The measurement slope of the LSV curves and onset potential (E_ONSET_) were determined from the figures.

## 3. Results

### 3.1. Characterization of Cu Nanocones

A cross-view of the nanocone template synthesized in oxalic acid after four cycles of the multi-step anodization and pore widening process is shown in Figure 1a. Based on the SEM images shown in Figure 1b,c, the intercone and interpore distances were measured and compared.

Figure 1a shows the obtained template, with the conical nanopores indicated by a red arrow. This confirmed that it is possible to synthesize a template with conical nanopores using two-step anodization. To check the quality of the prepared 1D conical templates, a copper deposition was performed under conditions taken from the earlier published work [17].

The measurements confirmed that it is possible to obtain templates with conical nanopores to produce free-standing nanostructures. The synthesized nanocones were characterized by a perfect match with the used template. The distance between the top part of the conical nanopores in the template and the intercone distance for free-standing Cu nanocones was about 70 nm in both cases. The dimension of the bases of the nanopores was about 116 nm, which corresponded with the actual cone shapes and size.

**Figure 2:**
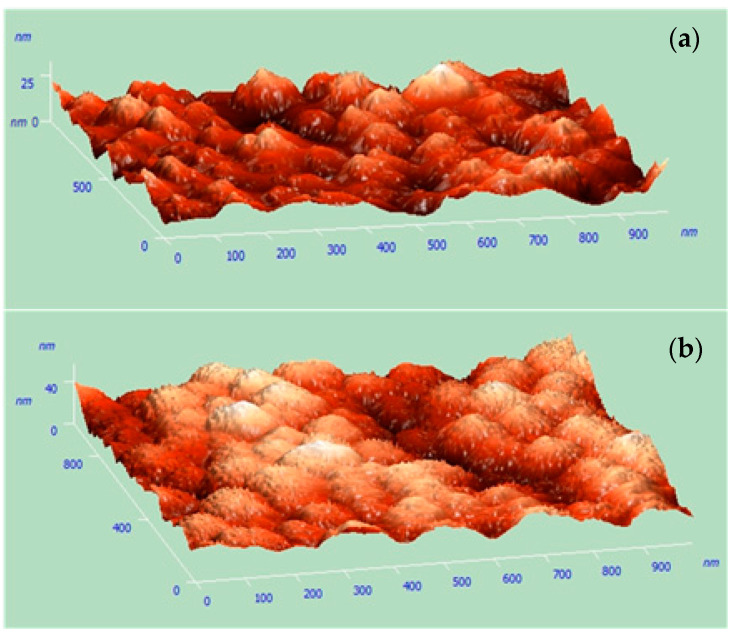
Atomic force microscope (AFM) morphology analysis of the produced nanocones: (**a**) central and (**b**) edge area of the sample.

To confirm the presence of the conical shape of the Cu nanostructures, the AFM measurements were performed. The digital representation of the surface topography is shown in Figure 2.

This analysis confirmed that copper nanocones were successfully obtained. The morphology was analyzed in different places on the sample surface. Some differences in the shape and height of the nanocones suggested that the nanocone template was of a better quality in the central part of the specimen. These structures were sharp-ended and conical ones.

### 3.2. Electrodeposition of Co–Fe Alloys

**Figure 3:**
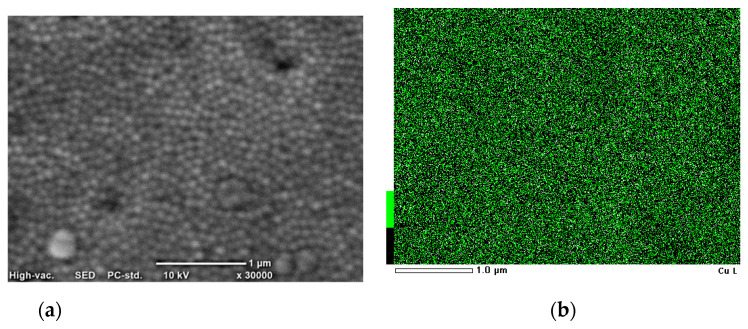
(**a**) SEM photo and (**b**) mapping analysis of the conical nanostructures’ synthesis in templates obtained in oxalic acid.

The distribution of metal was analyzed using the EDS method. Figure 3 shows the results with SEM images.

The EDS analysis confirmed that copper nanocones with a uniform distribution of Cu were obtained via electrodeposition.

Finally, Co–Fe bulk coating and Co–Fe nanoconical structures were electrodeposited in potentiostatic measurements. The obtained coatings were analyzed to determine their quantitative compositions using the X-ray fluorescence (XRF) method and to compare microstructures using SEM. These results with a mapping analysis are shown in Table 1.

SEM images, mapping analysis and the chemical compositions of Co–Fe bulk alloys electrodeposited from two different electrolytes are available in Figure S1 in the Supplementary Material.

The electrodeposition of Co–Fe alloy from these electrolytes is possible. The obtained coatings showed the different concentrations of Co and Fe and different morphologies, which depended on the solution used. The distributions of Co and Fe on these alloy surfaces were uniform. The last step was the synthesis of Co–Fe nanocones for 120 min at −1.285 V vs. the SCE from the first electrolyte.

### 3.3. Synthesis of Co–Fe Nanocones

**Figure 4:**
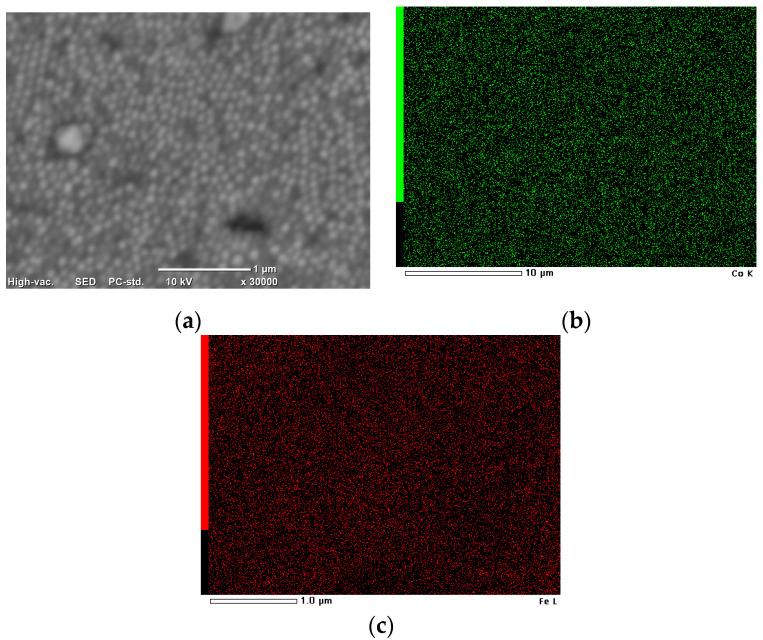
(**a**) SEM images and mapping analysis of the Co–Fe nanocones for (**b**) Co and (**c**) Fe.

SEM images of the obtained nanocones and the mapping analysis are shown in Figure 4.

The distribution of Co and Fe on the surface was uniform for the fabricated nanostructures.

**Figure 5:**
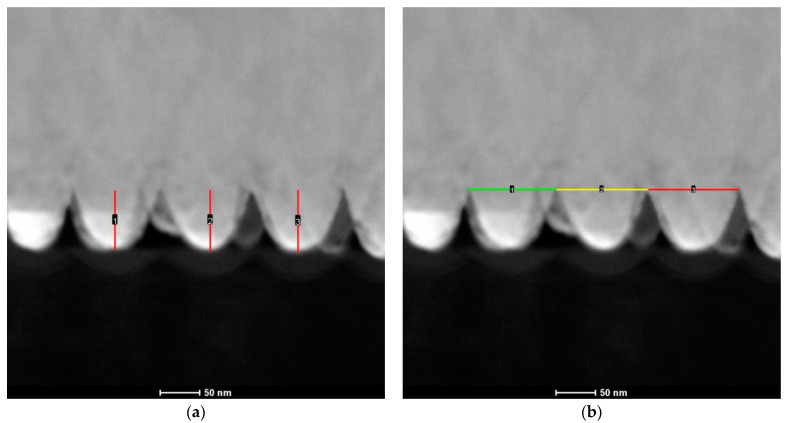
The comparison of the nanopore’s (**a**) average height (73.50 ± 1.04 nm) and (**b**) diameter (110.44 ± 1.84 nm) values.

The height and diameter of the nanocones were determined using a cross-section of Co–Fe nanostructures. The cross-sectional view was obtained using transmission electron microscopy (TEM) with a focused ion beam (FIB). The results are shown in Figure 5. These values were used to calculate the active surface of the sample.

The value of the calculated pore diameter based on the TEM photos was similar to that of the copper nanocones. The templates in both cases were prepared in 0.3 M H_2_C_2_O_4_. The value of the pore diameter and the height of the nanocones were averaged and used to calculate the active surface area. The size of the nanocones equaled 73.5 ± 1.0 nm, and the diameter was 110.4 ± 1.8 nm. Based on these values and the knowledge that there were 58 nanocones per 1 µm^2^ of the sample, the active surface area was calculated and was around 1.64 cm^2^.

**Figure 6:**
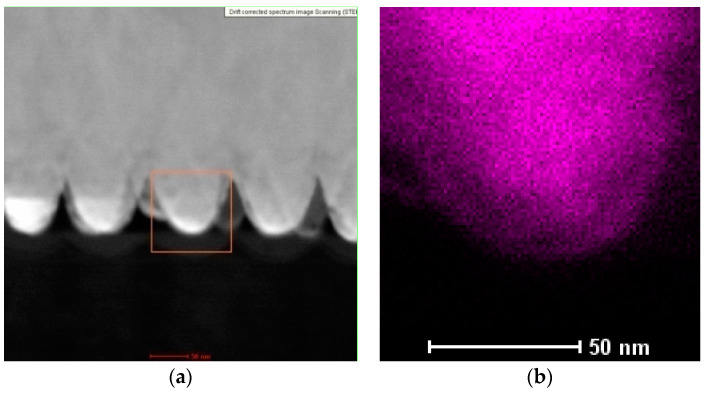

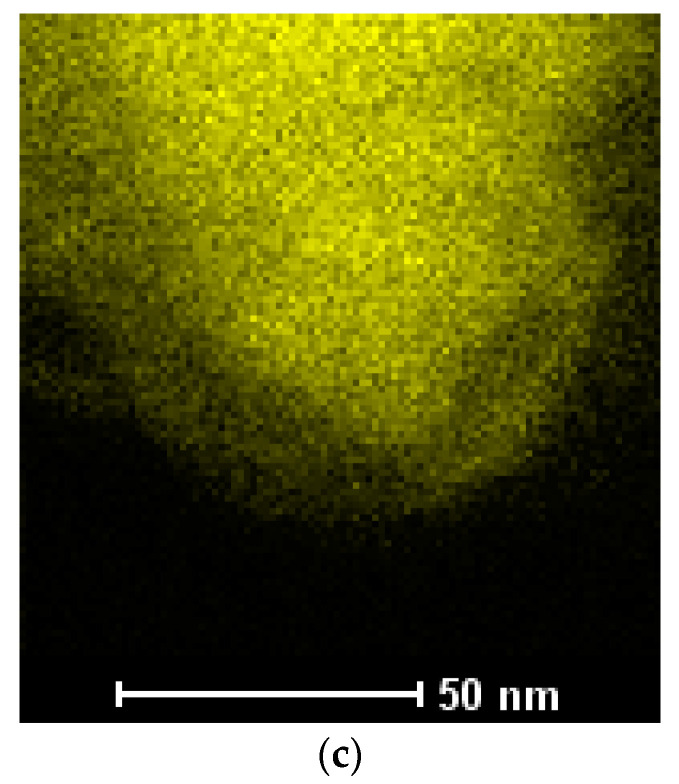
(**a**) TEM images of Co–Fe nanocones and distributions of (**b**) Co and (**c**) Fe.

Mapping analysis of a cross-sectional view of the Co–Fe nanocones was performed using EDAX. The TEM images and analysis results are shown in Figure 6.

The results confirmed that it was possible to obtain Co–Fe nanocones by electrodeposition in an alumina oxide template with the uniform distribution of Co and Fe in the pores.

Structural and morphological characterization using XRD and High-resolution transmission electron microscopy HR-TEM will be part of further studies. It is necessary to analyze the possible influence of coating morphology and structure on the filling of the nanopores and the distribution of elements.

### 3.4. Catalytic Examination of Nanocones in a Hydrogen Evolution Reaction

**Figure 7:**
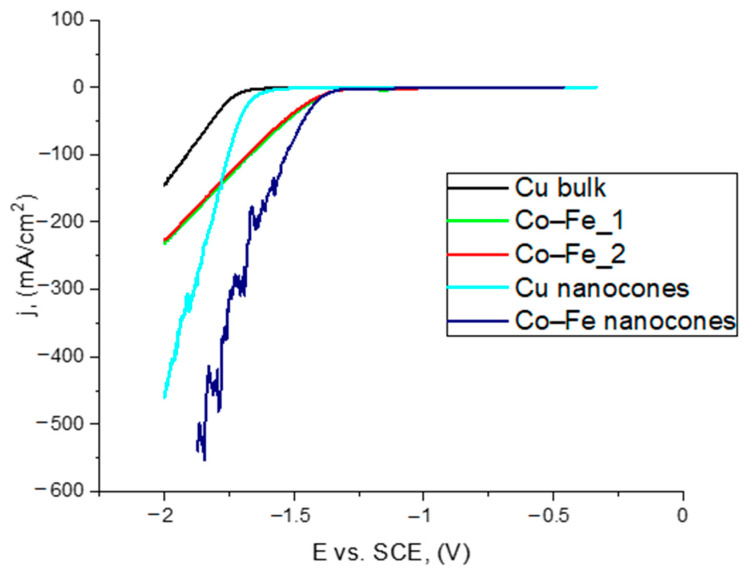
Linear sweep voltammetry (LSV) curves of Co–Fe alloys, Cu bulk and nanocone materials in a 1 M NaOH solution.

The electrocatalytic activity of the synthesized nanocones was determined. A comparison of the linear sweep voltammetry (LSV) curves of the fabricated materials is shown in Figure 7. The current density was estimated per unit of the calculated, active surface.

The Co–Fe nanocones showed the best LSV curve slope compared with Cu bulk and nanocones, as well as the Co–Fe alloys. The sharp character of the curve for the Co–Fe nanocones was connected with hydrogen bubbles appearing on the surface of the samples. They blocked this area. The possible behavior of hydrogen bubbles on the nanocones’ surface was described by using Cu and Co nanocones as examples [48].

**Figure 8:**
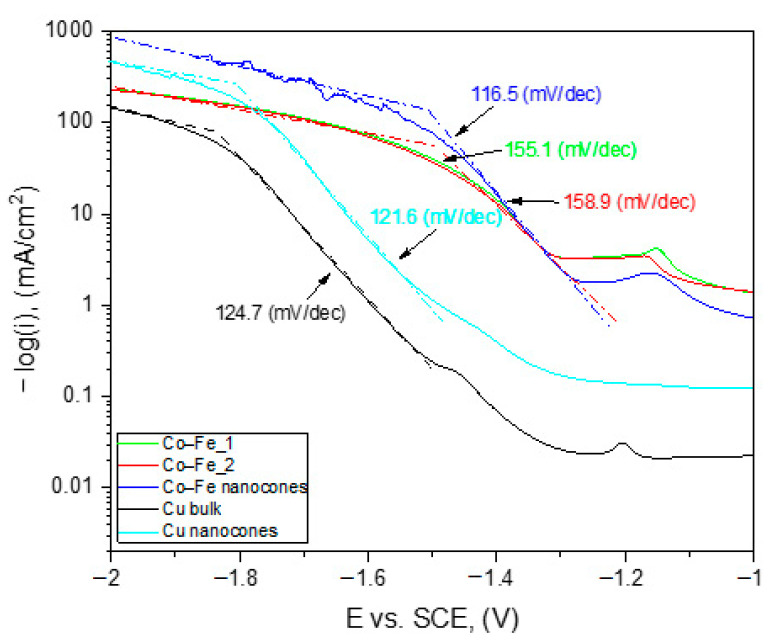
Tafel slopes of synthesized materials.

Tafel slopes were estimated for all tested materials. They are shown in Figure 8.

The results show that the materials showed different behavior of the Tafel slope. The biggest difference could be noticed in the case of the Co–Fe and Co–Fe nanocones, where an increment of the slope for low current densities decreased from 158 mV/dec for the Co–Fe alloy to 118 mV/dec for the Co–Fe nanoconical structures.

The obtained results were compared with the literature values. The results are shown in Table 2.

It can be found that Co was characterized by average catalytic activity (145 mV/dec). In the case of the iron electrodes, the hydrogen overpotential value was lower than in the cobalt, but the corrosion resistance in an alkaline environment was weaker. Synthesis of the Co–Fe alloy should have increased the stability and corrosion resistance of the electrode, which should exhibit good catalytic activity. The addition of C from lysine in the Co–Fe alloys and Co–Ni–Fe alloys with a small amount of Fe enhanced the activity and durability of electrodes for the evolution of hydrogen.

In the performed research, we observed that the nanoconical structures exhibited lower overpotential values of hydrogen evolution, due to the fact that the beginning of the water-splitting process could be observed for less negative potentials than for the bulk electrodes. Moreover, classical electrodes exhibited the higher value of the Tafel slope in both cases (Cu electrodes and Co–Fe). In the literature, there were no works connected with the catalytic activity of nanoconical alloys, like in the presented work.

Additionally, the values of E_ONSET_ and the values of the calculated active area were determined. The results are shown in Table 3.

For the Co–Fe nanocones, the hydrogen evolution reaction (HER) started earlier than for the Cu nanocones and copper bulk. The synthesized Cu and Co–Fe nanostructures had a larger active area than the other materials.

## 4. Conclusions

Based on the results of the experiments, the following was found:

- It is possible to get nanocones of pure Cu, which was confirmed by mapping analysis. There was no influence from the acid used on the distribution of Cu;
- The copper nanocones showed better electrocatalytic activity than the copper bulk. This was connected with the larger active surface area;
- Co–Fe alloys with different compositions and morphologies were successfully electrodeposited;
- The Co–Fe nanocones were obtained by electrodeposition on templates. The obtained alloy was characterized by a uniform distribution of Co and Fe;
- The nanostructures increased the active surface area of the electrodes;
- The Co–Fe nanocones showed the best electrocatalytic properties. The LSV curve had a sharp character, which may have been caused by the evolution of hydrogen and the active area being blocked by hydrogen bubbles. However, there was no significant increase in the electrocatalytic properties for the Co–Fe nanostructures compared with the bulk materials.

## Figures and Tables

**Table 1:** Chemical compositions of Co–Fe bulk alloys’ synthesis in templates obtained in various electrolytes.

Sample Name	Electrolyte Composition (mM)	Mass Co in Deposit (%)	Mass Fe in Deposit (%)
Co–Fe_1	6.5 CoSO_4_, 1.6 FeSO_4_ and 98.4 Na_2_SO_4_	81.90	18.10
Co–Fe_2	6.5 CoSO_4_, 3.3 FeSO_4_ and 96.8 Na_2_SO_4_	67.11	32.89

**Table 2:** Electrolytic properties of electrodeposited Co and Co-based alloys in alkaline solutions.

Material	Overpotential (mV/dec)	Solution (M)	Temperature (°C)	References
Co	145	8 NaOH	90	[49]
Co–Fe–C	35	8 NaOH	90	[50]
Co–Ni–Fe–C	36	8 NaOH	90	[51]

**Table 3:** Values of E_ONSET_ and the calculated area for the materials.

Material	E_ONSET_ (V)	Calculated Active Area (cm^2^)
Cu bulk	−1.72	1.20
Cu nanocones obtained in 0.3 M H_2_C_2_O_4_	−1.69	1.64
Co–Fe_1	−1.39	1.20
Co–Fe_2	−1.42	1.20
Co–Fe nanocones	−1.41	1.64

## Data Availability

Data is contained within the article and supplementary material.

## Supplementary Materials

The following are available online at https://www.mdpi.com/article/10.3390/ma14071717/s1. Figure S1: SEM images and mapping analysis and chemical composition of Co-Fe bulk alloys synthesized from electrolytes containing: (**a**) 6.5 mM CoSO_4_, 1.6 mM FeSO_4_ and 98.4 mM Na_2_SO_4_ and (**b**) 6.5 mM CoSO_4_, 3.3 mM FeSO_4_ and 96.8 mM Na_2_SO_4_.
