# Supplementary material for: Synthesis of Co–Fe 1D Nanocone Array Electrodes Using Aluminum Oxide Template
Source: Materials (Basel). 2021 Mar 31;14(7):1717. doi: 10.3390/ma14071717 (PMC8036432; doi:10.3390/ma14071717)
Supplement: Supplementary file 1 [file materials-14-01717-s001.pdf]

materials

MDPI

Supplementary Material

Synthesis of Co–Fe 1D Nanocone Array Electrodes Using Aluminum Oxide Template

Katarzyna Skibińska [1,*], Karolina Kołczyk-Siedlecka [1], Dawid Kutyła [1], Marta Gajewska [2] and Piotr Żabiński [1]

[1] Department of Physiochemistry and Metallurgy of Non-Ferrous Metals, Faculty of Non-Ferrous Metals, AGH University of Science and Technology, A. Mickiewicza 30, 30-059, Krakow, Poland; kkolczyk@agh.edu.pl (K.K.-S.), kutyla@agh.edu.pl (D.K.), zabinski@agh.edu.pl (P.Z.)

[2] Academic Centre for Materials and Nanotechnology, AGH University of Science and Technology, A. Mickiewicza 30, 30-059 Krakow, Poland; marta.gajewska@agh.edu.pl

* Correspondence: kskib@agh.edu.pl

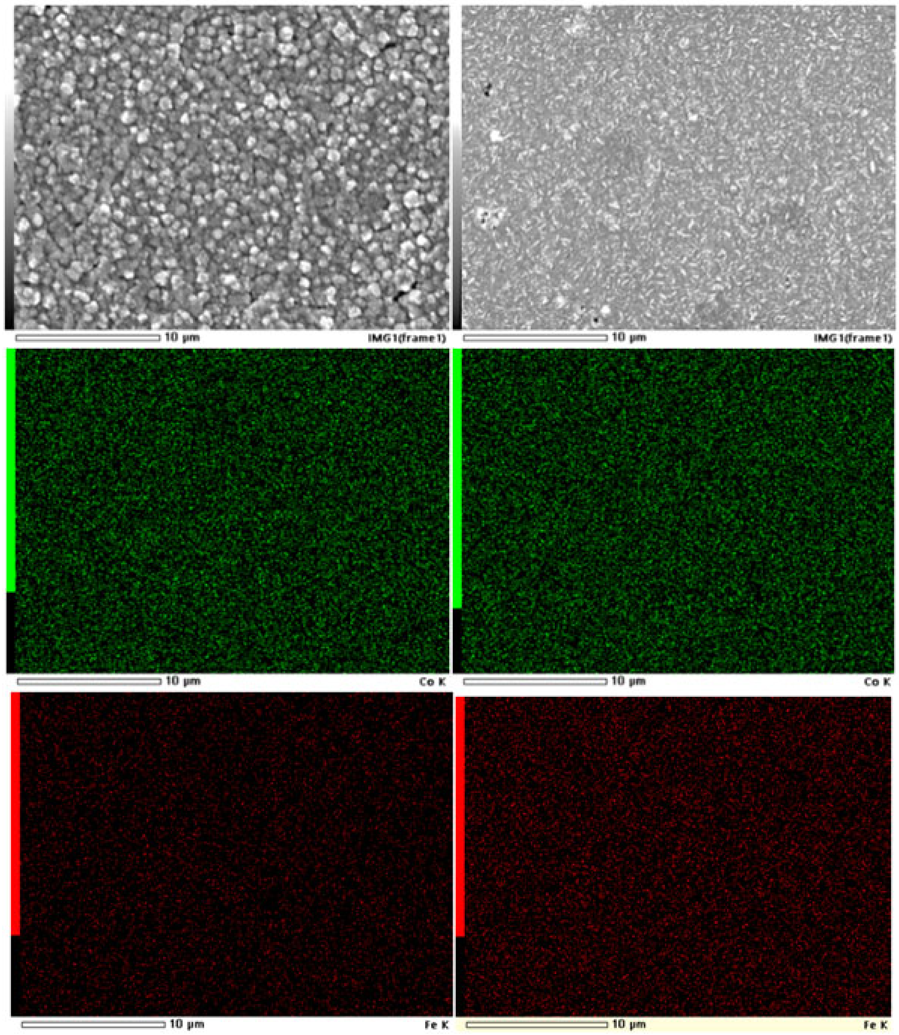

(**a**) (**b**)

Figure S1. SEM images, mapping analysis and chemical compositions of Co–Fe bulk alloys, synthesized from electrolytes containing (**a**) 6.5 mM $CoSO_4$, 1.6 mM $FeSO_4$ and 98.4 mM Na_2SO_4 and (**b**) 6.5 mM $CoSO_4$, 3.3 mM $FeSO_4$ and 96.8 mM Na_2SO_4.
